# Gas6/AXL pathway: immunological landscape and therapeutic potential

**DOI:** 10.3389/fonc.2023.1121130

**Published:** 2023-05-10

**Authors:** Xiaoqian Zhai, Dan Pu, Rulan Wang, Jiabi Zhang, Yiyun Lin, Yuqing Wang, Ni Zhai, Xuan Peng, Qinghua Zhou, Lu Li

**Affiliations:** ^1^ Department of Medical Oncology, Cancer Center, West China Hospital, Sichuan University, Chengdu, Sichuan, China; ^2^ Lung Cancer Center, West China Hospital, Sichuan University, Chengdu, Sichuan, China; ^3^ Department of Nutrition and Integrative Physiology, College of Health, University of Utah, Salt Lake City, UT, United States; ^4^ Graduate School of Biomedical Sciences, MD Anderson Cancer Center UT Health, Houston, TX, United States; ^5^ Graduate School of Biomedical Sciences, Baylor College of Medicine, Houston, TX, United States; ^6^ Neurosurgery Intensive Care Unit, The 987th Hospital of the Joint Logistics Support Force of the Chinese People's Liberation Army, Baoji, Shanxi, China; ^7^ Department of Pathophysiology, Hubei Minzu University, Enshi, Hubei, China

**Keywords:** Gas6/TAM, AXL, cancer development, TME, drug resistance

## Abstract

Cancer is a disease with ecological and evolutionary unity, which seriously affects the survival and quality of human beings. Currently, many reports have suggested Gas6 plays an important role in cancer. Binding of gas6 to TAM receptors is associated with the carcinogenetic mechanisms of multiple malignancies, such as in breast cancer, chronic lymphocytic leukemia, non-small cell lung cancer, melanoma, prostate cancer, etc., and shortened overall survival. It is accepted that the Gas6/TAM pathway can promote the malignant transformation of various types of cancer cells. Gas6 has the highest affinity for Axl, an important member of the TAM receptor family. Knockdown of the TAM receptors Axl significantly affects cell cycle progression in tumor cells. Interestingly, Gas6 also has an essential function in the tumor microenvironment. The Gas6/AXL pathway regulates angiogenesis, immune-related molecular markers and the secretion of certain cytokines in the tumor microenvironment, and also modulates the functions of a variety of immune cells. In addition, evidence suggests that the Gas6/AXL pathway is involved in tumor therapy resistance. Recently, multiple studies have begun to explore in depth the importance of the Gas6/AXL pathway as a potential tumor therapeutic target as well as its broad promise in immunotherapy; therefore, a timely review of the characteristics of the Gas6/AXL pathway and its value in tumor treatment strategies is warranted. This comprehensive review assessed the roles of Gas6 and AXL receptors and their associated pathways in carcinogenesis and cancer progression, summarized the impact of Gas6/AXL on the tumor microenvironment, and highlighted the recent research progress on the relationship between Gas6/AXL and cancer drug resistance.

## 1 Introduction

Cancer is a disease with ecological and evolutionary unity, which seriously affects the survival and quality of human beings (1). Cancer cells are described as invasive species and its metastasis as a multidirectional ecological dispersal. The foundational ecological principles include intraspecific relationship (e.g communication) and interspecific relationship (e.g competition, predation, parasitism and mutualism) are interpreted to understand cancer progression. In this review, we will mainly introduce the role of the Gas/AXL signaling in cancer cells, discuss its interaction with the tumor immune microenvironment, and its relationship with tumor progression.

Currently, many reports have suggested TAM receptors and ligands play an important role in cancer. TAM receptor family members include Tyro3, Axl, MerTK (collectively known as TAM) (2). The TAM ligand family includes human growth inhibitor specific 6 (GAS6), PROS1, LGALS3, Tulp-1 etc (3). Typical of these ligands include GAS6 and PROS1. GAS6 activates all members of the TAM receptor family, including AXL, while PROS1 activates only Tyro3 and MerTK (4). GAS6 gene expresses the Gas6 protein, a vitamin K-binding protein, originally reported to be upregulated in fibroblasts induced by growth inhibition under serum starvation conditions (5, 6), and can activate AXL in a concentration-dependent manner (7), showing the highest affinity for the Axl among all the TAM receptor family (8, 9). Gas6 binds to AXL, to regulate cell survival (10–13), promote tumor cell proliferation and migration, induce epithelial mesenchymal transition (EMT), inhibit tumor cell apoptosis, and participate in tumor stem cell maintenance (14–18) by activating multiple downstream pathways including JAK/STAT3 (19), PI3K/AKT/mTOR (20), Grb2/RAS/MEK/ERK1/2 (21) and FAK/Src/NF kappa B (22). In addition, Gas6/AXL shapes the suppressive tumor immune microenvironment by modulating angiogenesis in the tumor microenvironment (23), regulates immune-related molecular markers and controls the secretion of certain cytokines (24), regulates the functions of multiple immune cells (25), and interacts with tumor cells, host immune cells, and abnormal physiological factors (26). In adult normal cells, such as normal brain tissue, hippocampus, heart and liver, AXL expression is relatively low (27, 28); however, in certain malignant cells such as breast cancer, chronic lymphocytic leukemia, non-small cell lung cancer, melanoma, and prostate cancer cells, AXL is abnormally overexpressed (29–40), promotes tumor progression and reduces overall survival. Therefore, AXL may constitute an important prognostic biomarker and a potential therapeutic target. With the current review, we discussed the roles of Gas6 and AXL receptors in carcinogenesis and tumor progression; then we summarized the effects of the Gas6/AXL axis on the TME; finally, we focused on recent progress on the relationship between Gas6/AXL and cancer therapeutic resistance, to provide novel directions for future experimental design and tumor treatment strategies.

## 2 Biological functions of Gas6/AXL

### 2.1 Gas6

The TAM ligand family includes human growth inhibitor specific 6 (GAS6), PROS1, LGALS3, Tulp-1 etc (3). Typical of these ligands include GAS6 and PROS1. GAS6 activates all members of the TAM receptor family, including AXL, while PROS1 activates only Tyro3 and MerTK (4). GAS6 is a vitamin K-dependent protein abundantly expressed in fibroblasts 3T3 cells induced by growth inhibition under serum starvation conditions (6). Sequencing of the Gas6 protein by Manfioletti and colleagues revealed Gas6 is a secreted protein containing 678 amino acids with a molecular weight of 75 kDa (5, 6). It consists of an N-terminal Gla structural domain with a disulfide bond-maintained loop behind it, and four epidermal growth factor (EGF)-like structural domains next to the loop (41, 42) and a sex hormone-binding globulin (SHBG)-like structural domain at the C-terminal.

### 2.2 TAM receptors, especially focusing on AXL

TAM is the receptor of Gas6. TAM binds to Gas ligands and exerts multiple effects in diverse cells (2). TAM receptors mainly regulate cell survival, mediate removal of apoptotic cells through phagocytes *via* non-inflammatory reactions, and affect natural killer cell differentiation and platelet aggregation etc. (29, 43–45). The TAM receptor family belongs to receptor tyrosine kinases (RTKs), consisting of the three receptors Axl, MerTK and Tyro3. Gas6 can activate AXL in a concentration-dependent manner. Furthermore, Gas6 shows the highest affinity for the Axl receptor in the TAM family, and it was reported that AXL as one of the receptors has 3-10 times higher affinity for Gas6 compared with the other two members (8).

AXL, firstly identified in 1991, is a 140 kDa protein. In adult normal cells, such as normal brain tissue, hippocampus, heart and liver, AXL expression is relatively low (27, 28), but AXL levels are abnormally high in many human cancers, including non-small cell lung cancer (NSCLC), esophageal cancer, glioblastoma, breast cancer and chronic lymphocytic leukemia (29–40), which is associated with reduced overall survival and enhanced disease progression. Some cancer models further revealed that AXL expression is related to tumor cell motility, metastasis, and invasion. Thus, AXL has great potential as a promising prognostic biomarker and therapeutic target.

TAM has a single hydrophobic transmembrane structural domain, comprising extracellular structural domains similar to intercellular adhesion molecules (ICAM) and vascular cell adhesion molecules (VCAM) (46), which contain the sequence of fibronectin and immunoglobulin (47), thus promoting cell aggregation through homophilic or heterophilic binding (48). In addition, TAM contains a tyrosine kinase structural domain, indicating this family of receptors have both the characteristics of an adhesion molecule and the activity of a tyrosine kinase. The Ig-like structural domain of the TAM receptor interacts with the laminin G-like domains of its ligand, thereby activating an intracellular signaling cascade that regulates many genes transcriptionally (46).

## 3 Role of Gas6/AXL signaling in tumorigenesis

**Figure 1:**
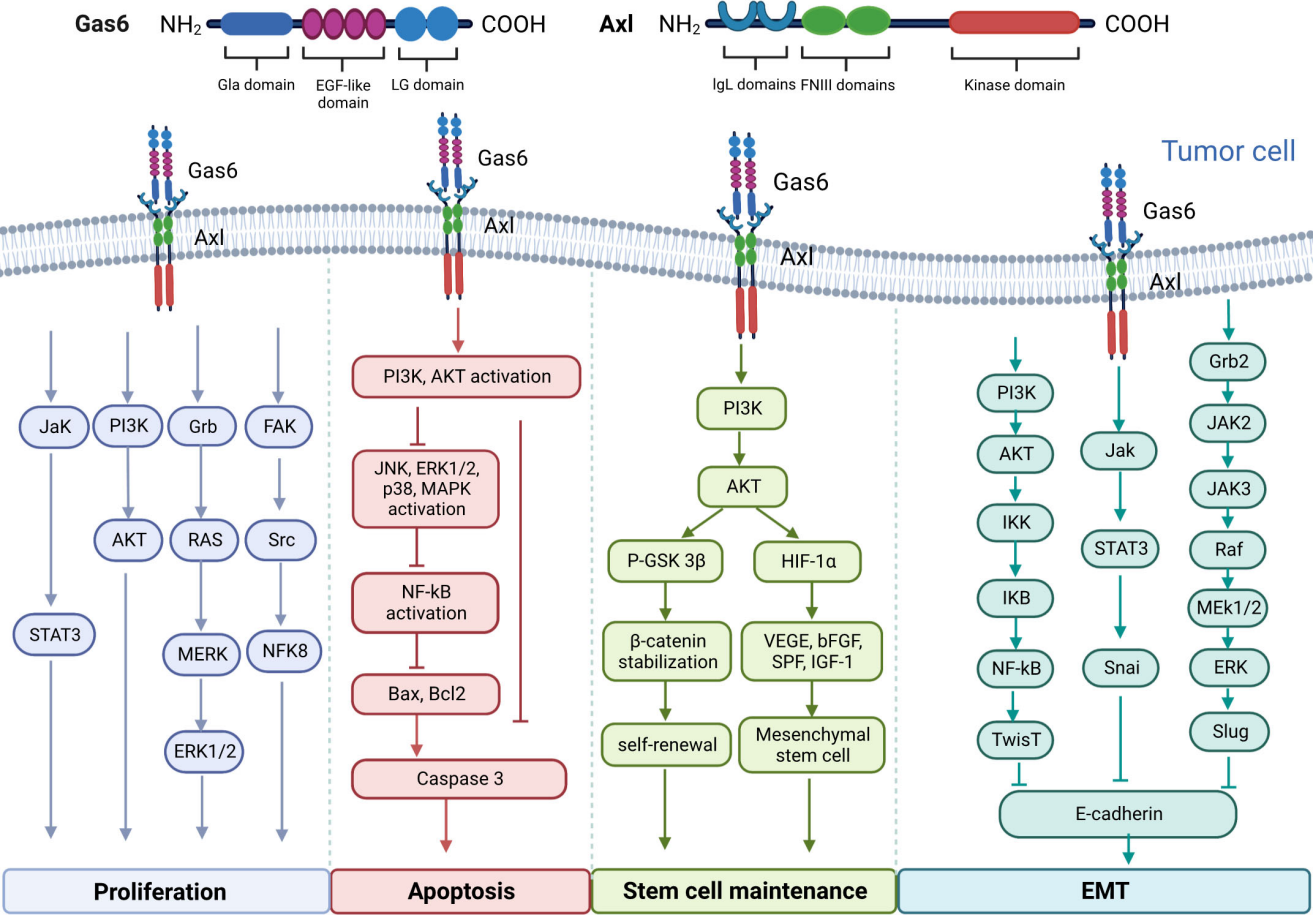
Molecular structure diagram of Gas6 and AXL and the multiple signaling pathways involved in the regulation of tumor cell proliferation, migration and apoptosis, as well as the maintenance of tumor stem cells by Gas6/AXL.

Upregulation of Gas6/AXL is associated with carcinogenesis in multiple malignancies and shortens overall survival, and may be involved in tumor cell proliferation, migration, apoptosis, and maintenance of tumor stem cells through multiple signaling pathways (**Figure 1**). Signaling pathways downstream Gas6/AXL signaling, including PI3K/AKT/mTOR, NF-κB, JAK/STAT3 and RAS/RAF/MEK/ERK, play critical roles in tumor cell cycle regulation, malignancy and drug resistance (14–17).

### 3.1 Gas6/AXL signaling axis promotes tumor cell proliferation

Post-binding activation of AXL and GAS6 is correlated with enhanced proliferation and survival in multiple tumors, including prostate, colorectal, gastric and renal cancers, and osteosarcoma (10–13). The main pathways controlling tumor cell proliferation induction include the STAT3 (19), PI3K/AKT (20), Grb2/RAS/MEK/ERK1/2 (21) and FAK/Src/NF kappa B pathways (22). Gas6 promotes the proliferation of AXL-expressing prostate cancer cells by enhancing Akt phosphorylation (49). Gas6 induces ERK signaling by interacting with AXL and promotes melanoma cell proliferation (50). Gas6/AXL signaling activates Src, local adhesion kinase (FAK) and NFκB to promote proliferation in nerve sheath tumor cells (22). In experimental studies of NSCLC and thyroid cancer, AXL silencing inhibited xenograft growth in nude mice (51, 52). In addition, it was shown cancer cells promote tumor growth by stimulating infiltrating leukocytes to express the mitogenic protein Gas6 (53).

### 3.2 Gas6/AXL signaling axis affects epithelial-to-mesenchymal transition

AXL is considered a driver of tumor metastasis. AXL activity highly contributes to the acquisition of migratory potential in cells (54). Tumor metastasis is tightly correlated with EMT. The intercellular adhesion of noncancerous epithelial cells contributes to maintaining tissue integrity; whereas mesenchymal cells migrate and invade (55). AXL activation drives EMT, suppresses the expression of epithelial biomarkers (e.g. E-calmodulin) and promotes the expression of mesenchymal biomarkers (e.g. N-calmodulin, Snail, Vimentin, Slug, α-catenin protein and α-SMA) (18) (36, 56–58),. Li and collaborators observed that Gas6 interaction with AXL induced tumor cell migration mostly by upregulating Slug in prostate and skin cancer cells (59). Yang and colleagues demonstrated AXL affects cell adhesion by phosphorylating myoglutinin on tyrosine in active myoglutinin filaments. This may indicate that AXL is involved in tumor cell migration (60). Similar findings have been reported in liposarcoma, and pancreatic, lung, breast and thyroid cancers (61–63). This further demonstrates an important role for the AXL pathway in tumor cell migration and invasion.

### 3.3 GAS6/AXL signaling inhibits apoptosis

Several reports have shown AXL’s association with suppressed apoptosis. The Gas6/AXL pathway represses apoptosis through PI3K/Akt pathway activation as well as *via* BAD (BCL2-associated cell death agonist) phosphorylation and ERK1/2 (64) activation. For example, Li et al, have shown that Axl is expressed in the cardiomyocytes in patients with sepsis, exogenous recombinant Gas6 can inhibit the activation of Axl/PI3K/Akt/NF-κB signaling pathway caused by bacterial infection, thereby inhibiting tumor necrosis factor (TNF)-a release and apoptosis, ameliorating sepsis-induced myocardial dysfunction (65). And it was found in tumor cells that the Axl-Gas6 receptor-ligand interaction can inhibit cell apoptosis and promote tumor progression by activating the AKT pathway and activating the NF-κB pathway (66, 67). In the TME, Gas6 can also inhibit apoptotic events in cultured VSMCs by phosphorylating AXL. Gas6 and AXL amounts increase upon vascular injury, playing a major role in neointima formation by inhibiting apoptosis (68). Under serum starvation conditions, acute myeloid leukemia (AML) Nomo-1 and Kasumi-1 cells with Gas6 and AXL silenced with two distinct shRNAs showed a two- to three-fold increase in apoptosis (69).

### 3.4 Gas6/AXL is associated with stem cell maintenance

Cancer stem cells can self-renew, differentiate, and become tumorigenic, which has a dramatic impact on tumor resistance, recurrence and metastasis (70). AXL correlates with many stem cell markers, including Isl1, Cdc2a, Bglap1, CD44 and ALDH1 (18). Gas6/AXL signaling stabilizes β-catenin through a p-AKT-dependent pathway thereby regulating the self-renewal capacity of leukemic stem cells (71). Gas6 can also enhance PI3K/AKT signaling through an AXL-dependent autocrine manner, thereby promoting factor-1 alpha (HIF-1α)-driven secretion of multiple growth factor-mediated maintenance of mesenchymal stem cells function (72).

## 4 Role of Gas6/AXL in the TME

**Figure 2:**
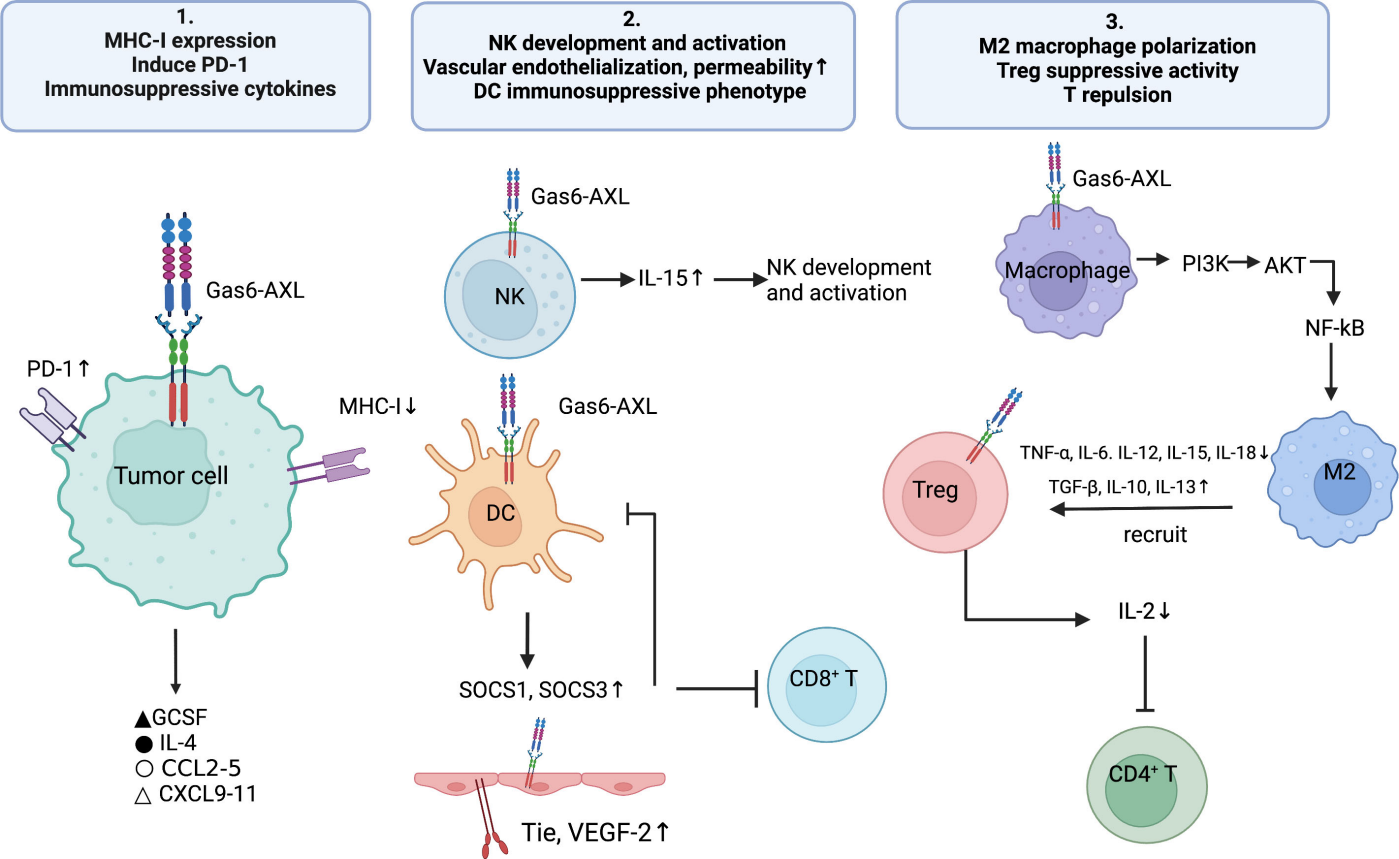
The Gas6/AXL pathway promotes the formation of an immunosuppressive microenvironment through multiple pathways: 1) regulating immune-related biomolecules, i.e., inhibiting the expression of MHC-I molecules and enhancing PD- L1 expression on tumor cells, promoting the secretion of immunosuppressive cytokines (e.g., IL-4, CCL3-5 and G-CSF) and inhibiting the secretion of chemokines that recruit Th1, CD8+ T cells and NK cells (CXCL9, CXCL10 and CXCL11); 2)enhancing the development and activation of NK cells; promoting the expression of Tie2 and VEGFR-2 on vascular endothelial cells, thereby inducing angiogenesis and reducing vascular permeability; enhancing the differentiation of DC cells to the immunosuppressive phenotype; 3) regulating immune cell functions, i.e., promoting M2-type polarization of macrophages, increasing the immunosuppressive activity of Treg cells and promoting T-cell repulsion.

The abovementioned findings described the cell-autonomous role of Gas6/AXL in malignant cells. The present section mainly summarizes the cell-dependent role of Gas6/AXL in the TME in malignant cells, e.g., the roles of immune and vascular smooth muscle cells (VSMC) on tumor development (**Figure 2**). The TME consists of tumor cells, tumor-supporting cells such as fibroblasts and vascular endothelial cells, secreted factors, and even impaired physiological conditions (73). In general, AXL is expressed on tumor cells, but recent reports detected AXL on bone marrow-derived cells (BMDC), dendritic cells (DC), giant phagocytosis cells, mononuclear cells, natural killers (NKs) and platelets (74). Myeloid cells may express AXL to apoptotic phagocytotic cells and debris. Additionally, tumor cells upregulate AXL and Gas6 expression in presence of monocyte myeloid-derived suppressor cells (M-MDSCs) and polymorphonuclear myeloid-derived suppressor cells (PMN-MDSCs) (26). Furthermore, hypoxia increases the expression of hypoxia-inducible factors-1 and -2 to upregulate genes conferring an aggressive tumor phenotype (75). Mounting evidence suggests hypoxic upregulation is tightly correlated with AXL expression and stability (76). Besides hypoxia, multiple cytokines upregulate AXL (77). Therefore, interactions among the tumor, host immune cells, and abnormal physiological factors in the TME may upregulate AXL and Gas6, thus promoting a pro-tumor microenvironment. Therefore, AXL might be a key mediator in the tumor malignant microenvironment.

### 4.1 The Role of Gas6/AXL signaling in angiogenesis

AXL is widely synthesized by endothelial cells and could promote angiogenesis by regulating the production of VEGF and PDGF, thus participating in the mediation of normal and tumor vascular systems. For instance, suppression of AXL reduces the expression of Tie2 and VEGFR-2 (78), leading to impaired vascular endotheliogenesis and affecting vascular permeability. Additionally, AXL suppression in xenograft-bearing mice decreases the endothelial cell marker CD31 in tumors (79) and reduces cancer cell-triggered angiogenesis. Downregulation of AXL or Gas6 can impair the formation and function of the vascular endothelium (80).

### 4.2 Gas6/AXL regulation of tumor immune response-related biomolecules

Substantial evidence shows GAS6/AXL signaling is important in the promotion of an immunosuppressive TME. The GAS6/AXL axis regulates many tumor immune-related biomolecules, e.g., major histocompatibility complex I (MHC-I) and programmed death ligand-1 (PD-L1) in tumor cells (24), and the levels of secreted anti-inflammatory cytokines such as IL-4, CCL3-5 and granulocyte colony-stimulating factor (G-CSF) (81). In addition, GAS6/AXL signaling also regulates the development and infiltration of several immune cells such as macrophages, DCs, NKs and regulatory T cells (Tregs), thus making it possible for tumors to evade immune surveillance.

#### 4.2.1 MHC-I

MHC-I molecules are found on all nucleated cells. Professional antigen presenting cells (APCs) lyse tumor cells by presenting MHC-I antigen epitopes to CD8^+^ T cells, inducing them to recognize tumor cells and secreting perforin and granzyme. Evidence indicates AXL is negatively associated with MHC-I molecules. Rothlin et al. found that TAM deficient mice have elevated *in vivo* amounts of MHC-I in myeloid cells, confirming for the first time an association between MHC-I and Axl (82). Jeon and co-workers demonstrated the AXL suppressor Q702 decreased the expression of TAM signature genes and upregulated MHC-I signature genes in tumor samples, and also reported upregulated CD8 T cell and NK cell signature genes in a time-dependent manner (83). Aguilera and collaborators suggested that in treatment unresponsive tumors, AXL was high expressed with antigen presentation suppressed through MHC-I, mediating immunological microenvironment reprogram and knockout of Axl in tumor cell lines increased surface MHC-I amounts in NF-κB independent pathway (84). Of more concern is the elevated AXL expression and the reduced expression of MHC class I molecules in the melanoma immunotherapy-resistant phenotype (85). In summary, there seems to be an extremely subtle relationship between AXL and MHC-I, which may be one of the mechanisms involved in immune evasion. But the exact regulation mechanism of how does AXL impact on MHC-I expression are still unclear, which deserves further exploration in subsequent studies.

#### 4.2.2 PD-L1

PD-L1 is produced by a variety of tumor cells, and its interaction with its receptor induces pathways for blocking T cell activation (86) to evade the host immune response (87). In cancer immunotherapy, TAM receptors play a key role in regulating immune checkpoint signaling associated with the PD-1 axis (88). In 2014, researchers demonstrated that activation of PtdSer-TAM-PD-L1-PI3k/Akt signaling in breast cancer promotes immune escape and chemotherapy resistance in tumors. A recent analysis showed increased expression of PD-L1 in HPV-negative head-and-neck squamous cell carcinoma (HNSCC) cells through AXL and PI3K signaling, which correlated with resistance to radiotherapy, causing local treatment failure and enhancing mortality in HNSCC (89). In lung adenocarcinoma PC9 and H1975 cells, pharmacological Axl inhibition with the selective Axl inhibitors bemcentinib and BGB324 markedly reduced PD-L1 and PD-L2 expression. In addition, in a preclinical model of breast cancer, combination of AXL suppression and anti-PD-1 resulted in both primary and metastatic tumor shrinkage, increased CD8T cell infiltration, and an increased rate of NK cell activation (90), which could not be achieved after treatment with either drug alone. Thus, AXL receptor kinase may affect the tumor immune microenvironment by regulating PD-L1 expression (91).

#### 4.2.3 Altered secretion

Gas6/AXL signaling promotes immunosuppression and generates a pro-tumor microenvironment by altering and regulating the secretion of cytokines associated with immune cell function and movement (92). G-CSF promotes granulocyte-myeloid-derived suppressor cell (G-MDSC) accumulation in the tumor immune microenvironment (93). Axl knockout results in reduced secretion of G-CSF (84). The selective Axl inhibitor bemcentinib reduces G-CSF secretion in pancreatic cancer animal models (94). Further data also showed pharmacological inhibition of Axl downregulates the pro-tumorigenic inflammatory factor IL-4 in tumors (95). Axl inhibition attenuates the secretion of chemokines (CCL 2-4 and CCL 5) involved in the recruitment of immunosuppressive cells, including M2 macrophages and MDSCs, but promotes the secretion of chemokines (CXCL9-11) enhancing the recruitment of immune-effector cells such as CD8^+^ T cells and NKs (96).

### 4.3 Gas6/TAM signaling regulates immune cell function

As Gas6/AXL signaling triggers an immunosuppressive tumor microenvironment, the functions of diverse immune cells and the overall makeup of the tumor immune microenvironment are modified in this process.

#### 4.3.1 The Gas6/AXL pathway regulates the functions of macrophages and dendritic cells

Tumor cells develop specific mechanisms, including efferocytosis, for removing apoptotic cells to regulate the immune response. AXL expression on specialized phagocytes (macrophages and dendritic cells) in various tumor settings is important for homeostasis under physiological conditions. The main mechanisms involve macrophage polarization and efferocytosis of apoptotic cells (43, 97).

TAM receptor-mediated efferocytosis has tumor-promoting functions such as immunosuppression, tumor metastasis, and resistance to therapeutics (25). Gas6/AXL removes apoptotic residues by activating efferocytosis in macrophages and DCs, an effect impaired in AXL-deficient mice (98). Activation of Axl and Mertk receptor kinases is essential for PtdSer-dependent efferocytosis of apoptotic cells. It was shown Axl- and Mertk-induced efferocytosis of apoptotic cells inhibits innate immune responses orchestrated by macrophages and DCs (99), thereby generating a TME that favors the formation of tumor development and metastasis (100). Chiu and colleagues found that in oral squamous cell carcinoma, AXL signaling controls the polarization of tumor-associated macrophages toward the M2 phenotype with elevated expression of M2 markers and genes (101). After TAM receptor-mediated efferocytosis and phagocytosis, tumor-associated macrophages tend to polarize toward the immunosuppressive phenotype (M2 macrophages) in response to various cytokines and suppress antitumor immunity (102). The underpinning mechanism might involve Axl/PI3K/Akt/NF-κB signaling, in which the TAM receptor binds directly to PI3K, leading to PI3K phosphorylation of Akt. This results in macrophage polarization toward the M2 phenotype while reducing the amounts of M1 macrophages (103).

Additionally, efferocytosis in turn upregulates TAM receptor in tumor APCs, making them polarize to an immunosuppressive phenotype (102). DCs have moderate AXL expression prior to encountering pathogens. However, after pathogen encounter, AXL is significantly overexpressed *via* strong induction of the JAK/STAT1 pathway (82), thus shifting the pro-inflammatory state to an immunosuppressive state (104). The shift in the APC phenotype leads to diminished T-cell antigen presentation, reduced T-cell activation, and impaired antigen-dependent antitumor immunity, generating a more aggressive and tolerant TME (8).

#### 4.3.2 The Gas6/AXL pathway regulates the activation of natural killer cells

The Gas6/AXL pathway plays a key role in the regulation of NK cell activity (105). It regulates the normal developmental process of NK cells and the function of killing infected cells (106) by controlling the expression of receptors necessary for NK cell activation (105). Several studies have shown that NK cell dysfunction is associated with tumor progression in multiple aspects, including immune evasion and tumor metastasis. Under hypoxic conditions, inhibition of NK cell function promotes the formation of pre-metastatic niches (107). Recombinant Gas6 and anti-AXL antibodies upregulate NK cell-specific receptors and NK cell-related genes (108), thereby promoting NK cell receptor activation. The cytotoxic function of NK cells was impaired in AXL-inactivated mice. Significantly less NK cells were produced by human CD34+ hematopoietic stem cells after blocking Gas6’s interaction with AXL by AXL-Fc or warfarin (106). In addition, interleukin 15 (IL-15), in case of AXL depletion, fails to induce multiple pathways necessary for NK cell development (2, 109). Thus, AXL is highly correlated with NK cell activation and function.

#### 4.3.3 The Gas6/AXL pathway promotes effector T cell exclusion

The physical contact of effector T cells with tumor cells is the basis for the efficacy of immunotherapy. Certain stromal cells in the TME present a state of an immune desert within the tumor by excluding T cells close to malignant cells (110). The main mechanisms include insufficient activation of DCs leading to blunted antigen presentation and the lack of tumor antigens in the TME to initiate the T-cell response (110).

The receptor tyrosine kinase (RTK) AXL may be a potential mediator of T-cell rejection, increasing tumor cell invasion and metastasis and suppressing the immune response by enhancing T-cell rejection (111). The mechanism appears to involve a role for AXL in the inhibition of antigen presentation and production of myeloid-supporting inflammatory molecules, which leads to an inadequate adaptive immunity and T-cell rejection (84). AXL inhibitors have immune activating and antitumor effects. In a previous study, CD4+ and CD8+ T cell amounts were significantly increased in tumor-bearing mice administered the AXL inhibitor R428 (96), corroborating Holtzhausen et al. (26). In transgenic mouse models, AXL gene deletion increases T-cell infiltration in the tumor microenvironment by up to 20 times, while making tumor cells 50 times more sensitive to radiotherapy and immune checkpoint therapy (112). A recent mouse study demonstrated that AXL inhibitors impact the immune status and tumor growth in lung cancer. Application of AXL inhibitors to treat mice resulted in delayed tumor growth, elevated rate of effector memory helper T cells, enhanced infiltration of central memory cytotoxic T cells, increased amounts of CD86+ macrophages, and elevated proportion of CD80 high-expression macrophages in the tumor model (113).

#### 4.3.4 The Gas6/AXL pathway regulates the immunosuppressive activity of Tregs

Regulatory T cells (Tregs) regulate immune evasion, considered the primary mechanism of evasion from immune surveillance (114). Tregs inhibit multiple physiological and pathological immune reactions, which are essential for maintaining self-tolerance and immune homeostasis (115). Gas6 enhances the inhibitory effect of Tregs mainly through the AXL receptor (23). The proliferative activity of T cells is obtained mainly *via* IL-2, a powerful growth factor. After GAS6 addition to a co-culture system comprising CD4+CD25-T cells and CD4+CD25+ Tregs, T-cell proliferation was reduced as well as IL-2 expression. After Axl knockout, Foxp3 expression in Tregs was decreased and IL-2 expression was increased. Therefore, Gas6 can inhibit CD4+ T cells by depleting IL-2 or inhibiting IL-2 production (116). These findings corroborated findings by Zhao and colleagues in mice (116).

## 5 Gas6/AXL signaling controls drug resistance in cancer

Tumor cell resistance is an important issue in cancer therapy, often leading to failed treatment or recurrent disease. Besides its roles in survival, proliferation and migration, AXL expression is a possible mechanism underlying resistance to immunotherapy, chemotherapy and molecularly targeted therapies. AXL may lead to innate or acquired resistance to chemotherapy, immunotherapy, molecularly targeted therapies and even radiotherapy (117, 118).

### 5.1 Gas6/AXL and chemotherapy resistance

Hong et al. found that chemotherapeutic agents such as etoposide (VP-16) and cisplatin induce AXL upregulation in resistant acute leukemia, as a potential mechanism of chemoresistance (38). Wang and collaborators demonstrated AXL’s involvement in breast cancer resistance to adriamycin. AXL inhibitors combined with adriamycin markedly decrease the tumor load as well as invasion and metastasis in adriamycin-resistant breast cancer (119). AXL was also reported in pancreatic ductal adenocarcinoma to promote resistance to chemotherapy (120). AXL mRNA amounts were significantly elevated in cisplatin-resistant ovarian cancer cells compared with cisplatin-sensitive cells (121).

### 5.2 Gas6/AXL and targeted therapy drug resistance

Widespread overexpression of AXL is also found in tumors following resistance to various targeted therapies (122, 123), resulting in cell tolerance or under-response to molecular targeted therapies such as EGFR, VEGFR, ALK, ERK, and PI3Kα inhibitors (79, 122). Inhibition of AXL, either by silencing or pharmacological intervention, effectively circumvents the resistance of targeted drug-resistant cell lines to certain targeted drugs. AXL overexpression and Kit downregulation were detected in imatinib-resistant gastrointestinal mesenchymal tumors, hence the term ‘‘tyrosine kinase switch’’ was coined for AXL (118). The same findings were reported in NSCLC models with resistance to erlotinib. Taniguchi et al. further showed that EGFR mutant NSCLC administered ostatinib had increased AXL expression, the extent of which was inversely correlated with the effect of ostatinib. The combination of AXL inhibitors increased sensitivity to ostatinib treatment compared with ostatinib monotherapy, both in primary and resistant cases, thereby reducing tumor size and slowing tumor growth (124). In addition, current evidence suggests that AXL overexpression modulates acquired resistance to cetuximab in NSCLC and HNSCC models (122).

### 5.3 Gas6/AXL and immunotherapy resistance

As described in Section 3 of this paper, the Gas6/AXL pathway regulates the immune microenvironment by modulating important components of the immune microenvironment, including the tumor’s vascular system; critical biomarkers such as MHC-I molecules and PD-L1; important cytokines such as IL-4, CCL3-5 and G-CSF; and key immune cells such as phagocytes, DCs, NK cells, effector T cells and Tregs. In addition, it induces the formation of an immunosuppressive microenvironment, suppresses the host’s antitumor immunity, and mediates tumor immune escape. Therefore, the relationship between Gas6/AXL signaling and resistance to immunotherapy deserves further attention. By comparing the transcriptomes of PD-1-responding and non-responding tumors, Hugo et al. found that AXL is upregulated in non-responding tumors (125) and may be one of the key genes mediating resistance to immunotherapy. The relationship between AXL and resistance to immunotherapy was further investigated by Aguilera and colleagues, who found that in Py8119 cells, a mouse breast cancer cell line expressing Axl, radiotherapy combined with immune checkpoint inhibitors induced a limited initial immune response, exhibiting an immunotherapy-resistant state. Py8119 cells with Axl knockdown (by the CRISPR technology) transplanted into naive C57Bl/6 mice showed sensitivity to immunotherapy, delayed tumor growth, increased expression of MHC-I molecules and enhanced infiltration of mature DCs, CD4+ T cells, and CD8+ T cells in the tumor tissue (84). Mechanistically, AXL-mediated immune resistance involves a complex molecular network of multiple pathways and targets. Targeting AXL to sensitize to immunotherapy is associated with multiple biological events, including MAPK inhibition, NF-κB activation, and ICAM1 and ULBP1 upregulation (126). Further studies should focus on validating these findings and exploring how AXL drives immune resistance.

The above evidence provides a theoretical basis for the development of AXL-related drugs in combination with conventional therapeutic modalities based on synthetic lethality in the context of tumor resistance to therapy. Given the role of AXL in cancer growth and metastasis as well as its relatively low expression in normal tissues compared with tumor tissues, AXL represents a highly potential therapeutic target in cancer therapy. Currently, a series of therapeutic drugs targeting AXL have been developed, including small molecule inhibitors (117, 127), monoclonal antibodies (mAbs), antibody-drug conjugates (ADCs) (128), soluble receptors (129), and chimeric antigen receptors (CARs) T Cells (130). Some of drugs show obvious anti-tumor activity (**Tables 1**, **2**). In addition, the potential of AXL inhibitors in combination with other anti-tumor therapies (especially checkpoint inhibition) has also received increasing attention. For example, multiple AXL drugs including bemcentinib, ONO-7475 (131), sitravantinib (127), mecbotamab vedotin (128), and batiraxcept (129) in combination with ICI for patients refractory to first-line immune drugs are currently in phase I-III trails (**Tables 1**, **2**). However, there are still many problems to be solved in the current research on AXL-related drugs, which need to be further clarified according to the research results.

**Table 1:** Clinical study of GAS6/AXL related drugs for cancer.

Target	Drug type	Drugs	Condition(s)	Phase	NCT no	Satus
AXL	ADCs	BA3011/CAB-AXL-ADC	NSCLC	2	NCT04681131	Recruiting
			Sarcoma	1/2	NCT03425279	Recruiting
			Ovarian Cancer	2	NCT04918186	Recruiting
AXL	Small molecules	Bemcentinib/BGB324/R428	MS	2	NCT03824080	Completed
			NSCLC	2	NCT03184571	Completed
			Breast Cancer	2	NCT03184558	Terminated
			NSCLC	1/2	NCT02424617	Completed
			AML/MS	1/2	NCT02488408	Active, not recruiting
			Glioblastoma	1	NCT03965494	Active, not recruiting
AXL	Small molecules	SCL-391	Solid Tumor	1	NCT03990454	Recruiting
AXL	mAbs	Tilvestamab/BGB149	Ovarian Neoplasms	1	NCT04893551	Recruiting
AXL	ADCs	Enapotamab vedotin	Solid Tumors	1/2	NCT02988817	Completed
AXL	mAbs	Mecbotamab vedotin/CABAXL-ADC	Advanced Solid Tumors	1/2	NCT03425279	Recruiting
	ADCs		NSCLC	2	NCT04681131	Recruiting
AXL	ADCs	Mipasetamab uzoptirine /ADCT-601	Advanced Solid Tumors	1	NCT05389462	Recruiting
Gas6/AXL	Small molecules	Batiraxcept/AVB-S6-500	Ovarian Cancer	1	NCT03639246	Completed
			Urothelial Carcinoma	1	NCT04004442	Active, not recruiting
			Ovarian Cancer	3	NCT04729608	Active, not recruiting
			Pancreatic Adenocarcinoma	1/2	NCT04983407	Recruiting
			CCRCC	1/2	NCT04300140	Active, not recruiting
			ovarian, fallopian tube, or primary peritoneal cancer	1/2	NCT04019288	Active, not recruiting
Axl, Met, RON, FLT3	Small molecules	BMS-777607/ASLAN002	Advanced Solid Tumors	1/2	NCT00605618	Completed
			Malignant Solid Tumour	1	NCT01721148	Completed
Axl, Met	Small molecules	BPI-9016 M	Solid Tumors	1	NCT02478866	Completed
Axl, MerTK		INCB081776	Solid Tumors	1	NCT03522142	Recruiting
Axl, Aurora A and B, JAK2, Alk, Abl, Mer	Small molecules	TP-0903/Dubermatinib	Advanced Solid Tumors	1	NCT02729298	Completed
			FLT3 Mutated AML	1	NCT04518345	Completed
Axl, Src kinases, Abl, TGF, BMP	ADCs	BA3011/CAB-AXL-ADC	NSCLC	2	NCT04681131	Recruiting
			Solid Tumor	2	NCT03425279	Recruiting
			Ovarian Cancer	2	NCT04918186	Recruiting
AXL, MER, TYRO3, VEGFR2, KIT, METAXL, MER	Small molecules	SNS-314	Advanced solid tumors	1	NCT00519662	Completed
Axl, MerTK	Small molecules	ONO-7475	Acute Leukemias	1/2	NCT03176277	Terminated
			Advanced or Metastatic Solid Tumors	1	NCT03730337	Suspended
AXL, MER, TYRO3, VEGFR2, KIT, MET	Small molecules	Sitravatinib (MGCD516)	CCRCC	2	NCT03680521	Active, not recruiting
			Metastatic Non-Squamous NSCLC	3	NCT03906071	Active, not recruiting
			NSCLC	2	NCT02954991	Completed
			Advanced Cancer	1	NCT02219711	Completed
			Advanced or Metastatic Kidney Cancer	1/2	NCT03015740	Completed
			CCRCC	1	NCT04518046	Active, not recruiting
			Urothelial Carcinoma	2	NCT03606174	Completed
			HCC/GC/GJC	1/2	NCT03941873	Active, not recruiting
			NSCLC	2	NCT02664935	Active, not recruiting

antibody-drug conjugates= ADCs; monoclonal antibodies= mAbs; Non-Small Cell Lung Cancer= NSCLC; Myelodysplastic Syndromes= MS; Acute Myeloid Leukemia= AML; Clear Cell Renal Cell Carcinoma= CCRCC; Hepatocellular Carcinoma= HCC; Gastric Cancer= GC; Gastroesophageal Junction Cancer= GJC.

**Table 2:** Published clinical studies of GAS6/AXL related drugs for cancer.

Target	Drugs	Condition(s)	Design	Phase	Clinical responses	NCT no	Satus	time of the latest results
Gas6	AVB-S6-500	Ovarian Cancer	A: AVB-S6-500+PLD B: AVB-S6-500+Pac	1b/2	A: ORR: 10.7%; mDoR:4.2 months; PFS: 3.6months; OS: 11.2months B: ORR: 34.8%; mDoR:7.0 months; PFS: 3.1months; OS: 10.3months	NCT03639246	Completed	2021
AXL, MER, TYRO3, VEGFR2, KIT, MET	Sitravatinib /MGCD516	CCRCC	Sitravatinib+nivolumab	1/2	ORR: 35.7%; PFS: 11.7months	NCT0301574	Completed	2022
		Advanced Cancer	Sitravatinib	1	ORR: 11.8%	NCT02219711	Completed	2022
		Liposarcoma	Sitravatinib	2	mPFS: 11.7 weeks; OS: 31.7 weeks	NCT02978859	Active, not recruiting	2023
		NSCLC	Sitravatinib+tislelizumab	1	ORR: 8.7%-57.1%	NCT03666143	Completed	2023

NSCLC, Non-Small Cell Lung Cancer; CCRCC, Clear Cell Renal Cell Carcinoma.

## 6 Problems and prospects

Roles for Gas6/AXL signaling in cancer development and progression, and shaping of the tumor microenvironment are gradually being revealed. Recently, substantial resources have been deployed to develop broad therapies targeting Gas6/AXL in cancer (132). Currently, cancer drug development strategies targeting AXL and other family members of its receptor include small-molecule inhibitors, monoclonal antibodies, and soluble receptors. Most of these studies focused on the exploration of small molecule inhibitors, while some reports focused on the regulation of its upstream effector Gas6. Among these drugs, BGB324 currently shows promising results in early studies of acute myeloid leukemia, based on its high specificity (133, 134). In addition, as mentioned above, a growing number of studies have started to examine the impact of AXL on conventional therapy, e.g., AXL inhibitors in combination with chemotherapy, targeted therapies, and especially immunotherapy, and these advances have increased our understanding of tumor biology, tumor progression, and the tumor immune microenvironment, showing promising prospect. However, the current analysis is still in the nascent stage and many questions remain unaddressed. For example, the mechanisms involved in the regulation of AXL in immunotherapy have not been fully elucidated. Future research should focus on preclinical determination of the optimal combination of cytotoxic and immunomodulatory therapies, initiation of innovative trials to assess the most promising combinations, and evaluation of the efficacy and toxicity of these therapies. The combination of anti-AXL therapies with chemotherapy and/or immunotherapy may represent an excellent opportunity.

## Author contributions

Guarantor of integrity of the entire study: XP, QZ and LL. Study conception and design: XP, QZ and LL. Literature search: XZ, DP, RW, JZ, YL, YW, NZ. Clinical studies: XZ, DP, RW, JZ, YL, YW, NZ. Experimental studies/data analysis: XZ, DP, RW, JZ, YL, YW, NZ. Statistical analysis: XZ, DP, RW, JZ, YL, YW, NZ. Manuscript preparation: All authors. Manuscript editing: All authors. All authors contributed to the article and approved the submitted version.
